# Combined Dielectric Spectroscopy and Operando DRIFTS Analysis of Ba-Based NO_x_ Storage Materials for Radio-Frequency-Based NO_x_ Dosimeters

**DOI:** 10.3390/s26103203

**Published:** 2026-05-19

**Authors:** Daniela Schönauer-Kamin, Fabian Fütterer, Johanna Baumgärtner, Thomas Wöhrl, Gunter Hagen, Ralf Moos

**Affiliations:** Department of Functional Materials, University of Bayreuth, Universitätsstraße 30, 95447 Bayreuth, Germany

**Keywords:** RF-based NO_x_ dosimeter, Pt/Ba–Al_2_O_3_, operando DRIFTS, microwave cavity perturbation, dielectric properties, nitrite/nitrate formation

## Abstract

**Highlights:**

**What are the main findings?**

- The dielectric losses (*ε*″) of a NO_x_ storage catalyst show a linear correlation with NO_x_ loading and Ba site utilization, making them the most reliable indicator of the NO_x_ storage state.
- DRIFTS and capacitance results confirm temperature-dependent formation of nitrites and nitrates.

**What are the implications of the main findings?**

- *ε*″ serves as a clearly interpretable signal for RF-based NO_x_ dosimeter sensors with Pt/Ba–Al_2_O_3_-based materials at 350 °C.
- The identified nitrate-dominated mechanisms enable targeted optimization of future RF transducers with respect to linearity and performance.

**Abstract:**

This study investigates the dielectric behavior and NO_x_ storage properties of Pt/Ba–Al_2_O_3_ NO_x_ storage materials using microwave cavity perturbation, operando DRIFTS, and impedance spectroscopy with respect to their applicability in a radio-frequency-based NO_x_ dosimeter-type sensor. Dielectric losses (*ε*″) are identified as the most sensitive indicator of NO_x_ storage, exhibiting a clear linear correlation with both the accumulated NO_x_ dose and the utilization of Ba storage sites. Approximately 35% of the available Ba sites participate in nitrite and nitrate formation, and the absolute dielectric loss response increases proportionally with the Ba content of the NOx storage catalyst. In contrast, the permittivity (*ε*′) shows only minor changes, which are mainly influenced by temperature. Temperature-dependent experiments reveal stable NO_x_ storage with negligible desorption up to 350 °C, whereas pronounced desorption processes at 400 °C significantly limit the linear dosimeter behavior. Operando DRIFTS measurements on Pt/Ba–Al_2_O_3_ functional films confirm temperature-dependent formation of nitrites and nitrates, with nitrates dominating the NO_x_ storage at elevated temperatures. Capacitance measurements show a slight increase during NO_x_ storage, indicating a moderate increase in permittivity. Overall, Pt/Ba–Al_2_O_3_ NO_x_ storage materials exhibit a robust, quantitatively interpretable dielectric response that is well suited for radio-frequency-based, dosimeter-type NO_x_ sensing.

## 1. Introduction

The accurate detection and quantification of nitrogen oxides (NO_x_) remain key requirements for emission monitoring in industrial and automotive applications and for air-quality monitoring. Nitrogen oxides pose significant risks to human health and the environment, with NO_2_ being particularly harmful, and regulatory frameworks focus strongly on limiting ambient NO_2_ concentrations. The European Union has therefore set an hourly limit of 200 µg m^−3^ (≈105 ppb) and an annual limit of 40 µg m^−3^ for NO_2_ (Directive 2008/50/EC) [1]. Robust and long-term stable sensor systems are becoming increasingly important for monitoring the limit values in order to comply with the permitted limits and accurately reflect real emission situations. Well-known NO_x_ detection systems, such as chemiluminescence detectors (CLDs), are highly accurate, but are costly, bulky, and require substantial maintenance. With classical gas sensing devices, long-term monitoring at low concentrations is complicated by zero-point drift, signal noise, and nonlinearities [2,3,4,5,6,7,8,9,10].

Gas dosimeters that rely on the accumulation of analytes in sensitive materials circumvent several of these challenges and offer a promising alternative to conventional gas sensing concepts when long-term integrative measurements are required [11]. Since gas dosimeters directly detect the amount or dose of an analyte species by summing up the concentration over an interval, low amounts and concentrations can be detected more precisely, particularly during long-term measurements. The dose of NO_x_ *n*_NOx_ is, in the case of a constant gas flow rate, calculated according to Equation (1) as the timely integral of the NO_x_ concentration *c*_NOx_ (*t*) with the starting point of the sorption phase *t*_0_. The dose *n*_NOx_ is often given in the unit ppm·h, which can be converted to mL if accounting for the applied gas flow rate:

(1) $$n_{{NO}_{x}}=\;\;\int_{t_{0}}^{t}c_{{NO}_{x}}\;dt.$$

For NO_x_ detection, many dosimeter concepts are based on measurable changes in the electrical resistivity of the sensing film due to sorption of NO_x_ molecules, such as potassium- or manganese-based materials [11]. Several studies have indicated the potential of impedimetric NO_x_ dosimeters. Groß et al. demonstrated that KMnO_4_-based impedimetric dosimeters enable linear monitoring of NO_x_ accumulation with periodic regeneration [12]. However, many NO_x_ storage materials exhibit low electrical conductivity, limiting the applicability of classical low-frequency impedance or resistive measurement techniques. Barium carbonate (BaCO_3_), for example, is an attractive NO_x_ storage material and well-known NO_x_ storage catalyst (NSC) [13,14,15]; however, BaCO_3_ exhibits an electrical conductivity of ~10^−11^ S cm^−1^, roughly six orders of magnitude lower than potassium carbonate (K_2_CO_3_) (~10^−5^ S cm^−1^), which itself requires stabilization due to hygroscopic sensitivity [16,17,18].

Radio frequency (RF) characterization has emerged as a powerful approach to overcome these limitations, as it enables the detection of changes in both the dielectric and conductive properties of a gas-sensitive material. RF sensing has proven effective, for instance, in determining the NO_x_ storage state of barium-based catalysts in exhaust gas applications [19]. Complementarily, planar microwave sensor concepts, such as microstrip- or stripline-based resonators, have enabled the development of compact gas sensors. In these sensors, the sensitive film perturbs the electromagnetic field, allowing the detection of analyte through shifts in resonant frequency or quality factor [20,21,22,23,24,25,26,27,28,29,30,31,32,33].

Linking RF signal formation to the underlying chemistry of NO_x_ storage materials is therefore essential for the development of robust RF-based NO_x_ dosimeters. Spectroscopic techniques such as Diffuse Reflectance Infrared Fourier Transformed Spectroscopy (DRIFTS) are well established for studying NO_x_ storage processes on catalytic materials [34,35,36,37,38]. DRIFTS is particularly valuable for identifying nitrate and nitrite species, monitoring their formation pathways, and gaining mechanistic insights into sorption and desorption processes under realistic conditions. Such analytical techniques, especially operando DRIFTS setups, provide essential additional information on the chemical state of NO_x_ storage materials and support the interpretation of electrical and dielectric sensor signals [39,40,41,42,43,44,45]. In an earlier operando DRIFTS study of a resistive NO_x_ dosimeter based on potassium- and manganese-containing NO_x_ storage materials, the formation of nitrates and nitrites was detected, while at the same time a change in the resistance of the NO_x_ storage film was observed as the formation of nitrite/nitrate species increased [39]. Understanding how the formation of nitrates and nitrites affects the dielectric properties is crucial for assessing their suitability as functional films in RF-based NO_x_ dosimeters.

This study investigates the dielectric properties of Pt–BaCO_3_–Al_2_O_3_ NO_x_ storage materials using the microwave cavity perturbation (MCP) method, enabling in-depth analysis of dielectric losses and permittivity during NO_x_ sorption and regeneration [46]. Complementary operando DRIFTS and impedance measurements on screen-printed storage films provide mechanistic insights into the surface chemistry and its correlation with the RF signal. The combined results establish a comprehensive understanding of the material’s dielectric response and form a basis for evaluating Ba-based NO_x_ storage materials for application in radio frequency NO_x_ dosimetry.

## 2. Materials and Methods

### 2.1. Preparation of NO_x_ Storage Materials

The barium-containing NO_x_ storage powders were prepared by impregnating an aluminum oxide support. Lanthanum-stabilized γ-aluminum oxide powder with a high specific surface area (Puralox SCFa-140 L3, Sasol Germany GmbH, Hamburg, Germany, purity > 99.7%) was used as support. An aqueous barium acetate Ba(CH_3_COO)_2_ (Merck KGaA, Darmstadt, Germany, purity > 99%) solution was infiltrated with different concentrations to achieve different Ba loading amounts. The impregnated powders were dried at 110 °C and calcined afterwards at 550 °C for four hours in air atmosphere. During the calcination process, barium carbonate, barium oxides and barium hydroxide were formed depending on the calcination atmosphere, which contained CO_2_, O_2_, and humidity. Three different barium carbonate loadings of 5.6 wt.%, 11.2 wt.%, and 16.9 wt.% (hereinafter denoted to as: NSC-Ba6, NSC-Ba11, and NSC-Ba17) were prepared. In the next step, platinum was added to each Ba-loaded powder to improve the oxidation of NO and achieve sensitivity for both NO_2_ and NO. Therefore, tetraamine platinum (II) chloride Pt(NH_3_)_4_Cl_2_ dissolved in water (Merck; pure platinum content of approx. 55 wt.%) was added to the powders with an amount of 1 wt.% relative to the mass of the barium-loaded powder. After drying, a reduction step at 500 °C in forming gas (5% H_2_ in N_2_) for two hours followed to achieve approximately 0.6 wt.% of elementary platinum [46,47]. Using this fabrication process, Pt/Ba–Al_2_O_3_-based NO_x_ storage materials with varying Ba contents were prepared and subsequently characterized.

### 2.2. Experimental Method for Determining Dielectric Properties Using the Microwave Cavity Perturbation Technique

The dielectric properties of the powdered barium-based NO_x_ storage materials were determined using the microwave cavity perturbation (MCP) method in a cavity resonator optimized for porous powder samples, following Dietrich et al. [48] and Steiner et al. [49] and previously described in [46]. In this technique, a cylindrical metallic cavity acts as a resonator into which electromagnetic waves are coupled, exciting defined resonance modes. The sample is placed inside a glass tube within the resonator, perturbing the electromagnetic field and causing a shift in the resonance frequency *f*_res_ and the quality factor *Q*. From these changes, the permittivity *ε*″ and the dielectric losses *ε*″ of the material can be derived according to Equations (2) and (3) (from [46]):

(2) $$\Delta f\;\sim \;(\varepsilon^{\prime }-1)\;\;,$$

(3) $$∆\frac{1}{Q}\sim \varepsilon^{\prime \prime }.$$

These correlations are only valid when the sample itself has a negligible influence on the electromagnetic field within the resonator. This condition is not met for samples with large volumes or high dielectric losses. Nonetheless, such perturbations can be compensated through established correction procedures. For porous samples, the intrinsic properties of the solid phase can be extracted by incorporating the influence of air on the effective permittivity, typically through the application of appropriate mixing models [46,50,51].

The used measurement setup, described in detail in the literature [46,48], allows temperature control up to 600 °C via an inline heater. The sample temperature was determined as the mean value of two type-K thermocouples that were placed up- and downstream of the powder sample. Furthermore, the NO_x_ storage powders inserted can be exposed to varying gas atmospheres. For NO_x_ storage measurements, a base gas of 20 vol.% O_2_ in N_2_ was used with a total flow rate of 500 mL/min, and defined concentrations of NO and NO_2_ were added. Meanwhile, the concentrations of NO and NO_2_ downstream of the NO_x_ storage material (and prior to the powder measurements with an empty setup) were analyzed by FTIR spectroscopy. Before and after each NO_x_ storage experiment, a thermal regeneration phase at 600 °C was initiated to release previously sorbed molecules, e.g., stored NO_x_.

For the investigations, 290 mg of NO_x_ storage powder was filled inside the glass tube of the MCP setup. Due to the high air content (≈90 vol.%) in the powder samples, effective material properties were estimated using the Wiener mixing rule. This assumes a linear relationship between the air fraction and the dielectric properties [52,53]. Although it remains uncertain whether this mixing rule is strictly applicable to the NO_x_ storage powders investigated, it nonetheless provides a consistent basis for comparing their NO_x_ storage and regeneration behavior, as all samples exhibited nearly identical air contents.

For radio frequency measurements during NO_x_ loading and regeneration phases, the scattering parameter *S*_21_ was measured using a vector network analyzer (VNA, MS46322B, Anritsu, Atsugi, Kanagawa Prefecture, Japan). The parameters of the resonance mode TM_010_ (located at a frequency of approximately 1.18 GHz) were analyzed and evaluated in terms of the resonant frequency *f*_res_ and quality factor *Q* using the methods described elsewhere [54,55,56]. Prior to each NO_x_ storage catalyst powder, a calibration measurement with an empty sample tube was performed. This step is necessary because the MCP method uses the resonant parameter shift caused by the insertion of the materials to determine their dielectric properties and to achieve a correction of the temperature dependency of the resonant frequency.

### 2.3. Operando DRIFTS Characterization of NO_x_ Storage Materials

In order to evaluate the NO_x_ storage powders with operando DRIFTS and simultaneous electrical characterization, a defined setup described in [39] was applied. The transducer consists of an alumina substrate with a screen-printed backside platinum heating structure, covered by a protective layer. With the four-wire platinum heating structure, measuring temperatures between 300 °C and 400 °C can be adjusted and the storage materials can be regenerated thermally at 600 °C. On the transducer’s top side, interdigitated gold electrodes with 70 µm line and 70 µm space (Au-IDE 70/70) are applied by screen-printing. The Au-IDE area is covered afterwards by a screen-printed NO_x_ storage film with film thicknesses in the range of 10 µm. Therefore, screen-printable pastes were prepared by mixing the NO_x_ storage powders with an organic binder system (mixture of ethyl cellulose and terpineol). After screen-printing and drying at 100 °C, the films were fired in air atmosphere at 650 °C for two hours.

The transducers were then assembled to the DRIFTS cell inside the FTIR spectrometer (Nicolet 6700 spectrometer, Thermo Fisher Scientific GmbH, Dreieich, Germany). The DRIFTS cell can be exposed to varying gas atmospheres with a flow rate of 500 mL/min, adjusted by mass flow controllers. Here, a base gas composition of 20% O_2_ in N_2_ was used and 2 ppm NO were added. In contrast to the studies conducted in the MCP setup, in which the analysis of NO_x_ adsorption of powders with NO_x_ concentrations of 400 ppm and 100 ppm, in some cases up to full NO_x_ loading, was carried out, the NO_x_ concentration was reduced for the investigation of NO_x_ adsorption processes using DRIFTS on porous screen-printed layers. The aim was to examine the range of interest for use as a dosimeter-type sensor, in which the relationship between the measured signal change and the NO_x_ dose is still linear. A higher NO_x_ concentration or dose would lead to rapid, complete loading of the film and thus reduce the signal resolution, particularly of the DRIFTS measurements.

During the gas exposure and resulting NO_x_ storage reactions on the NO_x_ storage films, the electrical properties of the NO_x_ storage films were measured by impedance spectroscopy (Novocontrol α-Analyzer, Novocontrol Technologies GmbH und Co. KG, Montabaur, Germany), and simultaneously, DRIFT spectra were obtained as single beam spectra every 1–2 min. For evaluation of both parameters, the sensors were first thermally regenerated at 600 °C in base gas and after cooling down to measuring temperature and a stabilization time, the measurements were started. After a certain time in base gas, the sensor was exposed to defined NO_x_ concentrations over 35 min, and the operando signals were measured simultaneously. Afterwards, a desorption phase (around 15–20 min) followed before starting the thermally initiated regeneration.

Initial impedance measurements were conducted in a frequency range of 1 MHz to 1 Hz with an effective voltage of 1 V during the NO_x_ sorption phase. The evaluation of the resulting Nyquist and Bode plots during NO_x_ loading indicates 948 kHz as a suitable measuring frequency for timely resolved investigations. The Bode plot showed capacitive and NO_x_-dependent behavior in the frequency range between 1 MHz and 1 kHz, with a phase angle of almost −90° and an inverse proportionality of the magnitude of the complex impedance |Z| with the frequency. It was checked beforehand that the recorded impedance signal did not vary with the effective voltage; hence, linearity was approved. From the measured magnitude of the complex impedance |*Z*|, the phase angle φ, and the frequency *f*, the capacitance *C* was calculated by Equation (4), considering a parallel *RC*-equivalent circuit [57,58]:

(4) $$C=\;\frac{\sin(-\varphi )}{2\;\pi \cdot f\cdot |Z|}.$$

From the obtained single beam DRIFTS spectra (intensity of the IR beam in a wavenumber range from 600 cm^−1^ to 4000 cm^−1^), the absorbance *A*, defined as the logarithm of the quotient of the intensities of the background spectra *I*_0_ (in base gas atmosphere at defined constant *T*) and the spectra during NO_x_ exposure *I* (NO_x_-containing atmosphere at same *T*), was calculated (Equation (5)):

(5) $$A=\log\frac{I_{0}}{I}.$$

## 3. Results and Discussion

### 3.1. Dielectric Properties of Pt/Ba-Based NO_x_ Storage Powders with Different Ba Loadings

**Figure 1:**
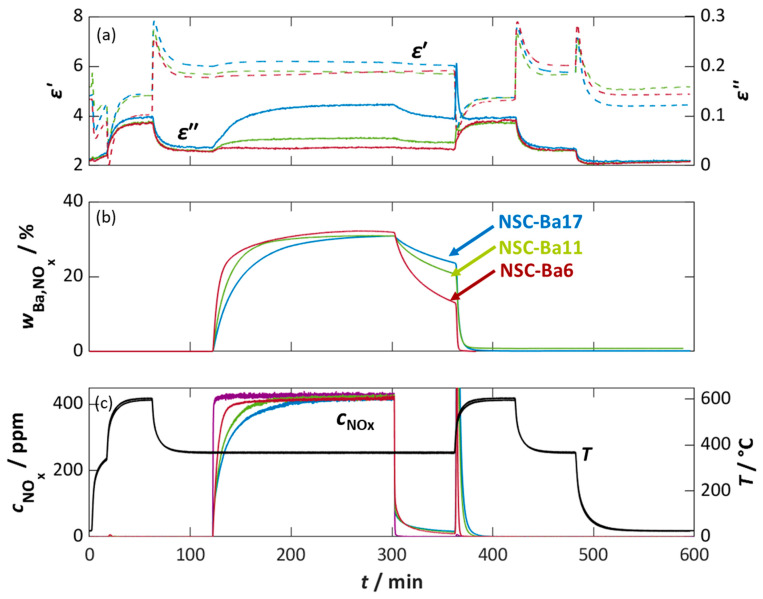
Dielectric parameters and NO_x_ storage behavior during NO_x_ sorption at 350 °C and regeneration at 600 °C on the barium content of the NO_x_ storage powders NSC-Ba6 (red), NSC-Ba11 (green), and NSC-Ba17 (blue). (**a**) Calculated permittivity *ε*′ (dashed lines) and dielectric losses *ε*″ (solid lines) of the NO_x_ storage materials, (**b**) calculated storage utilization *w*_Ba,NOx_ based on the integration of the measured nitrogen oxide concentration difference and the available Ba sites in each powder (Equation (6)), and (**c**) NO_x_ concentration *c*_NOx_ without (purple curve) and with the storage materials measured downstream of the MCP setup by FTIR as well as the temperature *T* of the storage material (black line).

The Pt-containing NO_x_ storage powders with different Ba loadings (NSC-Ba6, NSC-Ba11, and NSC-Ba17) were examined in the MCP setup to evaluate both their dielectric behavior during NO_x_ sorption and their NO_x_ storage properties (Figure 1). Prior to each experiment, the samples were thermally regenerated at 600 °C in base gas (see course of *T* in Figure 1c) and subsequently cooled to 350 °C. After a 60 min stabilization phase, 400 ppm NO_2_ was introduced at *t* = 120 min. The downstream NO_x_ concentration was monitored via FTIR and a reference measurement without powder (purple line in Figure 1c) was conducted. After 180 min, the NO_x_ dosing was stopped. A desorption phase at measuring temperature for 60 min followed, and then the samples were thermally regenerated at 600 °C to release sorbed NO_x_ species and cool down to 350 °C again.

The reference measurement without powder shows a stable NO_2_ dosing of 400 ppm (*c*_NOx,reference_) and does not show any desorbed NO_x_ species. The NO_x_ concentration profiles (*c*_NOx,NSC_) of the different storage materials differ markedly (Figure 1c). NSC-Ba17 shows the highest NO_x_ storage, as indicated by the lowest downstream NO_x_ concentration and the slowest increase to 400 ppm (blue line).

With decreasing Ba content, at 11.2% Ba (NSC-Ba11, green line) and 5.6% Ba (NSC-Ba6, red line), the measured NO_x_ concentration value increases more quickly. The time required to reach 400 ppm NO_x_ downstream increases with increasing Ba content. At this point, the catalyst material is fully loaded with NO_x_. The powder with the lowest Ba content (NSC-Ba6) reaches the reference NO_x_ concentration first, since a lower NO_x_ amount is necessary to completely load this powder. The stored amount of NO_x_, which corresponds to the difference in the area between the integrated reference NO_x_ concentration curve (purple curve in Figure 1c) and the integrated NSC–NO_x_ concentration curves, increases with Ba loading. This confirms that higher Ba contents provide a higher storage capacity. After all storage powders are fully loaded, a 60 min holding phase in base gas results in a small detectable NO_x_ release, almost independent of Ba content. This desorption is attributed to weakly bound NO_x_ species (e.g., physiosorbed species), resulting in a decreased NO_x_ loading level. During subsequent thermal regeneration at 600 °C (*t* = 360 min), all stored NO_x_ species are released, producing a pronounced NO_x_ peak. The area under the NO_x_ peak increases with increasing Ba loading. This is in good agreement with the NO_x_ storage capacity of the powders increasing with the content of barium [59,60,61,62,63].

From the resulting NO_x_ concentration difference during NO_x_ storage, the Ba site utilization *w*_Ba,NOx_ was calculated according to Equation (6) and as described in [46]. It indicates what proportion of the available barium carbonate molecules (related to the respective Ba content of each powder *n*_BaCO3_) was converted to barium nitrate during NO_x_ storage conditions. Assuming that each BaCO_3_ molecule is converted to barium nitrate (Ba(NO_3_)_2_) by binding two NO_x_ molecules, this results in a factor of two. Here, ${\dot{V}}_{gas}$ is the total volume flow and $V_{mol,gas}$ is the molar volume of the gas:

(6) $$w_{{Ba,NO}_{x}}=\int_{t_{0}}^{t}\left(c_{{NO}_{x,reference}\;}(t\right)-c_{{NO}_{x,NSC}\;}(t))\cdot \frac{{\dot{V}}_{gas}\left(t\right)}{V_{mol,\;gas}}\;dt\cdot \frac{1}{2\cdot n_{{BaCO}_{3}}}.$$

As shown in Figure 1b, all materials reach a similar maximum Ba site utilization of approximately 35% in the fully loaded state, regardless of their absolute Ba content. This indicates that only about one third of the available BaCO_3_ participates in nitrate formation under the applied conditions, which agrees well with the literature [60,61,62,63,64]. After stopping the dosing of NO_x_ (*t* = 300 min), *w*_Ba,NOx_ decreases due to the desorption of weakly bound NO_x_ species. The decrease is more pronounced for low Ba loadings, where a larger fraction of NO_x_ is weakly stored on the alumina surface rather than on Ba sites; an effect that is also well-known for NO_x_ storage catalysts [60].

The dielectric properties of the storage materials during NO_x_ sorption at 350 °C, derived from MCP measurements, are shown in Figure 1a. From the recorded resonant frequency *f*_res_ and the inverse quality factor 1/*Q*, the permittivity *ε*′ and the dielectric losses *ε*″ were calculated. As reported previously [46], the permittivity cannot be determined with high accuracy, and only a slight increase in *ε*′ is observed during NO_x_ exposure (starting at *t* = 120 min). However, the dielectric losses *ε*″ provide a more appropriate signal.

Depending on the barium loading, the dielectric losses increase from approximately 0.028 and 0.038 (NSC-Ba6 and NSC-Ba11, and NSC-Ba17, respectively) in the unloaded state to a maximum of 0.13 for the fully loaded NSC-Ba17. For lower Ba loadings, lower dielectric losses in the fully loaded state are determined (0.055 for NSC-Ba11 and 0.038 for NSC-Ba6). Especially for NSC-Ba17 and NSC-Ba11, *ε*″ increases significantly in the first third of the storage phase and approaches a stable final value as the materials become saturated with NO_x_. A stable value of *ε*″ is expected as soon as the catalyst material is fully loaded with NO_x_ and if no desorption reactions occur. A comparison of Figure 1a,c shows that the final value of the dielectric losses coincides with the point at which the downstream NO_x_ concentration reaches the reference value *c*_NOx,reference_. For example, NSC-Ba11 is fully loaded at *t* = 200 min, reflected by both the downstream concentration and the stabilization of *ε*″ at 0.055. The relative changes in dielectric losses are moderate, since NO_x_ sorption results in increases in the dielectric losses by a factor of 1.2 (NSC-Ba6) to 3.3 (NSC-Ba17). After NO_x_ dosing is stopped (*t* = 300 min), desorption of weakly bound NO_x_ species causes a slight decrease in *ε*″, predominantly due to the desorption of NO_x_ species from alumina sites. During thermal regeneration (*t* = 360 min), *ε*″ increases due to enhanced conductivity at elevated temperatures and returns to the pre-NO_x_ loading value after cooling down to 350 °C.

**Figure 2:**
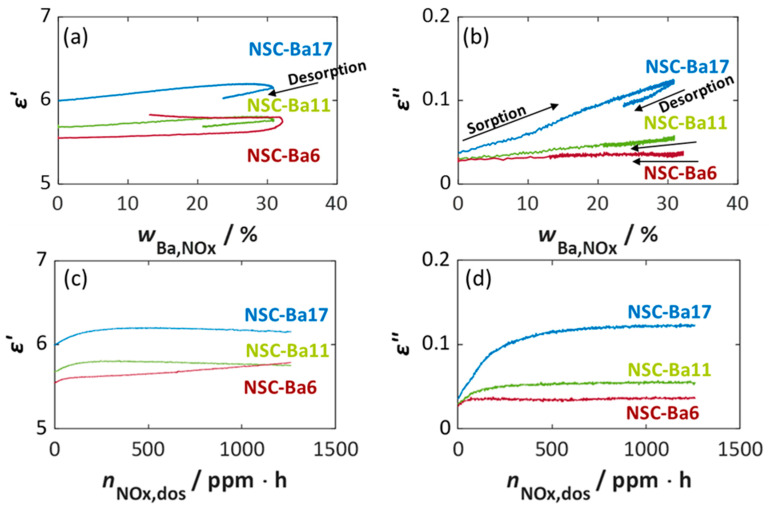
Permittivity *ε*′ (**a**,**c**) and dielectric losses *ε*″ (**b**,**d**) of the NO_x_ storage materials (NSC-Ba17 (blue), NSC-Ba11 (green), and NSC-Ba6 (red)) over the calculated storage utilization *w*_Ba,NOx_ (**a**,**b**) and dependent on the dosed amount of NO_x_ *n*_NOx,dos_ in ppm·h (dose of NO_x_) (**b**,**d**) at 350 °C.

A more detailed correlation between dielectric properties and both the storage utilization *w*_Ba,NOx_ and the dosed NO_x_ amount *n*_NOx,dos_ at 350 °C is shown in Figure 2. As already indicated in Figure 1, the permittivity *ε*′ (Figure 2a,c) exhibits only minor changes during NO_x_ storage. Although a weakly increasing trend with storage utilization can be observed, no meaningful dependence on Ba loading or NO_x_ dose can be derived. For this reason, *ε*′ is not considered further.

In contrast, the dielectric losses *ε*″ (Figure 2b,d) show a clear and pronounced dependency on Ba loading and consistently display a linear correlation with *w*_Ba,NOx_ (Figure 2b). Since only approximately 35% of the available Ba storage sites are converted to nitrite or nitrate under the applied NO_x_ storing conditions, the linear increase in dielectric losses scales with the fraction of Ba involved in NO_x_ storage, resulting in a varying sensitivity, defined as the slope of the curves [64]. The increase in *ε*″ can be attributed to the higher electrical conductivity of barium nitrites and nitrates compared to the initial barium carbonate or oxide phases [16,19,46,65]. For pure barium nitrate powder, significantly higher dielectric losses of approx. 0.4 at 350 °C have been reported [46].

The slope of the characteristic curves, meaning the increasing sensitivity, should ideally correspond to the content of barium in the materials. NSC-Ba6, containing roughly one third of the Ba present in NSC-Ba17, also proportionally forms a lower number of nitrate/nitrite species, although the same percentage of barium, approximately 35%, is used for NO_x_ storage [64]. Therefore, NSC-Ba6 shows a lower absolute increase in *ε*″ and ideally, the slope should be one third of that of NSC-Ba17. Deviations can be attributed to integration uncertainties and the small absolute changes in dielectric losses at low Ba loadings.

Figure 2a,b also show the desorption phase after the NO_x_ dosing was switched off. The decrease in *w*_Ba,NOx_, which can be seen for all NSC materials, is also evident in a measurable decrease in dielectric losses. This corresponds very well with the storage process, i.e., the curves overlap. This confirms that the decrease in dielectric losses directly reflects the decrease in *w*_Ba,NOx_ due to the release of weakly bound NO_x_ species [62]. With increasing Ba content, the desorption becomes less pronounced, i.e., with higher Ba content, the proportion of weakly sorbed nitrogen oxide species is lower. In accordance with the literature [60,61,62], both the binding strength of the NO_x_ species and the temperature stability increase with increasing Ba content, as the nitrogen oxides are preferentially sorbed at existing barium storage sites and the proportion of nitrogen oxides weakly sorbed on the Al_2_O_3_ surface (lower binding strength) thus decreases.

To evaluate the suitability of *ε*″ as a signal for dosimeter-type NO_x_ sensing, the dielectric losses were plotted against the dosed amount of nitrogen oxides *n*_NOx,dos_ in ppm·h, i.e., the NO_x_ dose (Figure 2d). At the onset of NO_x_ exposure, a linear relationship is observed, with *ε*″ increasing proportionally to the NO_x_ dose. This is particularly evident for NSC-Ba17 and NSC-Ba11, while NSC-Ba6 shows only a small increase in *ε*″. Once the maximum NO_x_ storage capacity of the material is reached, the dielectric losses remain constant and reach an almost stable value. At this NO_x_ dose, which is characteristic for each Ba content, the storage material is completely loaded with NO_x_ (see FTIR data in Figure 1c) and no further NO_x_ storage takes place, even if the NO_x_ dose will be increased. The final value of *ε*″ therefore corresponds to the maximum achievable value of the dielectric losses and correlates with the number of nitrates and nitrites formed or the number of occupied storage sites, e.g., occupied barium sites. For a RF-based dosimeter-type sensor, the linear region, in which *ε*″ correlates linearly with the NO_x_ dose, is the most relevant operational range. Assuming that in this region the sorption–desorption equilibrium is fully on the sorption side, all dosed NO_x_ is stored, and no desorption occurs during NO_x_ shut-off phases, the dielectric losses *ε*″ provide a reliable cumulative measure of NO_x_ dose.

**Figure 3:**
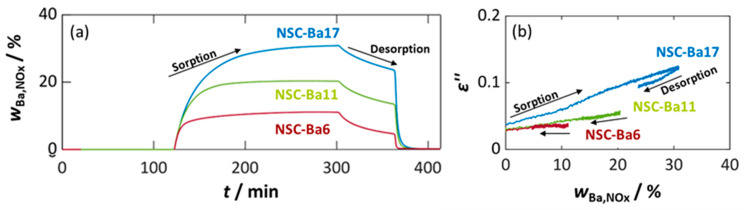
(**a**) Utilized amount of barium sites *w*_Ba,NOx_, calculated normalized to the maximum available amount of Ba in NSC-Ba17 during NO_x_ storage (data from Figure 1b) and (**b**) resulting dielectric losses *ε*″ versus the normalized storage utilization *w*_Ba,NOx_ (data from Figure 2b).

To highlight the influence of the Ba content more clearly, the normalized storage utilization *w*_Ba,NOx_ was recalculated using a different reference value of available barium carbonate molecules. Unlike in Figure 2, the reference value *n*_BaCO3_ used for normalization (Equation (6)) in Figure 3 was the maximum Ba content of 16.9% in the NSC-Ba17 material for the NO_x_ storage materials under investigation.

As shown in Figure 3a, the proportion of available barium sites converted to nitrates increases systematically with Ba content. NSC-Ba17 reaches about 35% utilization of the Ba sites at the end of the storage phase (see Figure 1b), whereas NSC-Ba6 reaches only 12%. As expected for different Ba loadings, the normalized storage utilization values differ by approximately a factor of three, consistent with the actual ratio of Ba contents and the corresponding number of available Ba storage sites. Assuming that the reaction rate of nitrate formation is independent of the absolute content of Ba [62], the total amount of nitrate species formed is directly proportional to the Ba content. Consequently, the dielectric losses correlate with the normalized storage utilization, as shown in Figure 3b. For all materials, *ε*″ increases linearly with *w*_Ba,NOx_, reflecting the progressive formation of nitrate and nitrite species, which exhibit significantly higher conductivity and thus higher dielectric losses than the initial carbonate phase. While an identical slope for all materials is expected, minor deviations occur that can be attributed to integration uncertainties (refer to Equation (1)). Additionally, relatively small absolute changes in dielectric losses are measured due to the limited storage capacity and the small amount of barium nitrate formed in comparison to the other materials (mostly Al_2_O_3_). Differences in *ε*″ in the unloaded state may also be due to the different material compositions. Nevertheless, a similar dependence of dielectric losses on storage utilization can be observed for different barium contents. Notably, for high Ba loading (NSC-Ba17), a well-defined linear relationship between *ε*″ and *w*_Ba,NOx_ can be derived, underscoring its suitability for a RF-based dosimeter-type sensor.

### 3.2. Temperature Dependency of the Dielectric Properties of Pt/Ba-Based NO_x_ Storage Powder NSC-Ba17

To further evaluate the suitability of NSC-Ba17 for RF-based dosimeter applications, the temperature-dependent behavior of its dielectric properties was investigated using a reduced NO_x_ dose of 150 ppm·h to examine the linear measuring range (see Figure 2). For this purpose, three pulses of 100 ppm NO or NO_2_ were added for 30 min each, separated by 30 min NO_x_ dosing pauses. The influence of temperature (300 °C, 350 °C, and 400 °C) and the difference between the addition of NO or NO_2_ were examined. NO_x_ loading was not investigated at temperatures exceeding 400 °C, since the literature consistently reports a decrease in NO_x_ storage capacity and a predominance of thermal desorption at higher temperatures [66,67]. Since dosimeter-type operation relies on an adsorption–desorption equilibrium shifted toward adsorption, temperatures above this range were therefore considered unsuitable.

**Figure 4:**
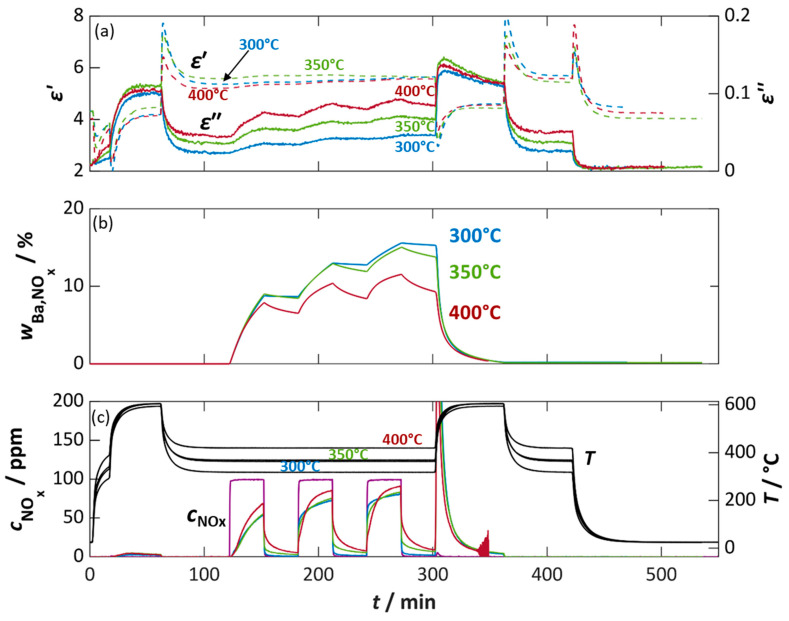
Dielectric parameters and NO_x_ storage behavior on NSC-Ba17 dependent on temperature (400 °C (red), 350 °C (green), and 300 °C (blue)) during pulse-like NO_x_ dosing of 100 ppm NO_2_ (three pulses, each 30 min): (**a**) permittivity ε′ (dashed lines) and dielectric losses ε″ (solid lines) of NSC-Ba17, (**b**) calculated storage utilization *w*_Ba,NOx_ based on the integration of the measured nitrogen oxide concentration and the available Ba sites, and (**c**) NO_x_ concentration *c*_NOx_ without (purple) and with the storage materials measured by FTIR downstream of the MCP setup, as well as the temperatures *T* of the storage material (black lines).

Figure 4 summarizes the resulting NO_x_ concentration (Figure 4c), dielectric parameters (Figure 4a), and calculated storage utilization (Figure 4b).

The reference curve in Figure 4c (purple curve) shows the added NO_2_ concentrations without powder in the MCP setup. The curves obtained for NSC-Ba17 at different temperatures reveal initial temperature-dependent differences in the storage capacity. During NO_2_ pulses, only minor differences can be detected, but during NO_2_ dosing pauses, more nitrogen oxides are desorbed as the temperature increases. During subsequent thermal desorption starting at *t* = 300 min, a clear short-term NO_x_ desorption peak occurs, indicating complete thermal regeneration. The calculated utilization of the Ba storage sites shows temperature-dependent differences in NO_2_ storage behavior (Figure 4b). Firstly, it is noticeable that *w*_Ba,NOx_ only reaches 16%, reflecting the lower amount of NO_2_ dosed (max. 150 ppm·h) compared to Figure 1b. The loading state of NSC-Ba17 is thus significantly lower. While NO_x_ storage at 300 °C and 350 °C follows an almost similar pattern, pronounced deviation occurs at 400 °C. The amount of NO_x_ stored (utilized amount of Ba) no longer increases linearly with time or NO_x_ dose. In addition, during NO_x_ dosing pauses, a significant desorption of NO_x_ is evident, resulting in a decreased *w*_Ba,NOx_. Consistent with the literature, the NO_x_ storage capacity decreases with increasing temperature [64]. At 300 °C, on the other hand, there is a linear relationship between the dose of NO_2_ and the proportion of Ba converted for all NO_x_ pulses. In the absence of NO_x_, no desorption processes take place, i.e., the sorbed nitrogen oxide species are strongly bound. These properties of the NO_x_ storage material are an essential requirement for dosimeter-type NO_x_ sensing [11]. At 350 °C, similar behavior can be observed until the second NO_2_ pulse, after which desorption effects become apparent, yielding deviations from linearity.

The dielectric properties of NSC-Ba17 presented in Figure 4a reflect these trends. As observed in Figure 1 and Figure 2, the permittivity *ε*′ changes only minorly. The dielectric losses *ε*″, however, closely follow the progression of *w*_Ba,NOx_, reflecting the formation of nitrite and nitrate species and the associated increase in electrical conductivity. With increasing measuring temperature, *ε*″ rises in the unloaded state due to its thermally enhanced electrical conductivity. Due to the sorption of NO_x_, almost linearly increasing dielectric losses are measured at 300 °C and 350 °C. At 400 °C, however, the increase in electrical losses is no longer linear and desorption effects result in a clear decrease in *ε*″. At 300 °C and 350 °C, *ε*″ remains stable during NO_x_ dosing pauses. Finally, the time-dependent dielectric losses *ε*″ behave dosimeter-like at temperatures below 400 °C.

The influence of temperature on the dielectric properties of the unloaded material becomes evident during regeneration at 600 °C compared to measurements at 300–400 °C (Figure 1 and Figure 4). In comparison to 400 °C, the evaluated permittivity is reduced at 600 °C, whereas no clear dependency can be identified between 300 °C and 400 °C, as discussed previously. In contrast, the dielectric losses *ε*″ exhibit a pronounced temperature dependence, with the highest losses observed at 600 °C and the lowest at 300 °C, or even at room temperature. This behavior is expected and can be attributed to the increase in electrical conductivity with an increase in temperature [16,19,68].

**Figure 5:**
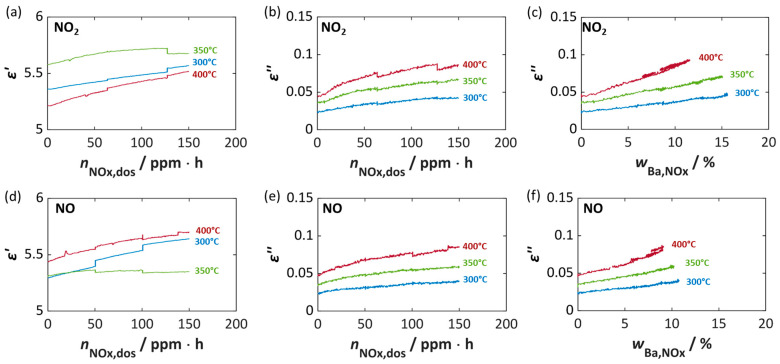
Permittivity *ε*′ (**a**,**d**) and dielectric losses *ε*″ (**b**,**c**,**e**,**f**) of NSC-Ba17 at different temperatures (400 °C (red), 350 °C (green), and 300 °C (blue)) over the dosed amount of NO_x_ *n*_NOx,dos_ during exposure to NO_2_ (**a**,**b**) and NO (**d**,**e**) and the dielectric losses *ε*″ over the calculated storage utilization *w*_Ba,NOx_ for NO_2_ and NO, respectively (**c**,**f**). The NO_x_ dose *n*_NOx,dos_, given in ppm·h, refers to the integrated concentration of NO_x_ (dose) at the total flow rate of 500 mL/min.

A more detailed interpretation is provided in Figure 5, in which *ε*′ and *ε*″ are plotted against the NO_x_ dose *n*_NOx,dos_ and the storage utilization *w*_Ba,NOx_ for different temperatures and NO_2_ or NO exposure. As discussed previously and in [46], no clear dependencies can be established for the permittivity *ε*′ owing to permanent temporal resonant frequency drift and strong temperature influences (NO_2_ Figure 5a; NO Figure 5d). In [46], a decrease in *ε*′ with increasing temperature was determined, whereas no clear dependence was measured here. It can be noted that ε′ is in the range of five to six and increases with NO_x_ dose, regardless of whether NO_2_ or NO was added.

In contrast, Figure 5b and Figure 5e confirm the dependence of the dielectric losses *ε*″ on the temperature and on the NO_x_ loading of NSC-Ba17. The increase in temperature results in an increase in dielectric losses in the unloaded state from approx. 0.022 (300 °C) to just under 0.048 (400 °C) (at *n*_NOx,dos_ = 0). With increasing NO_x_ dose and associated nitrate/nitrite formation, *ε*″ increases approximately linearly. At an accumulated dose of 150 ppm·h, NO_2_ exposure yields an increase in *ε*″ by a factor of 1.9, while NO results in a factor of 1.7, with only a slight temperature dependency. Desorption effects become more pronounced at higher temperatures, clearly reflected in the decreasing *ε*″ during NO_x_ dosing pauses (deviations in the figures at defined *n*_NOx,dos_ values).

**Figure 6:**
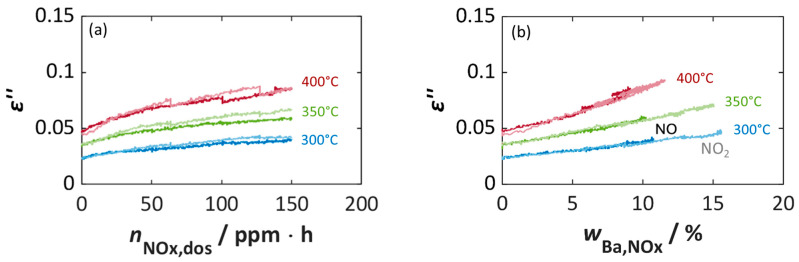
Dielectric losses *ε*″ of NSC-Ba17 during steplike NO (bold color tone) and NO_2_ (pale color) storage at different temperatures over (**a**) dosed NO_x_ amount *n*_NOx,dos_ and (**b**) the calculated NO_x_ storage utilization *w*_Ba,NOx_ (derived from data in Figure 5). The NO_x_ dose *n*_NOx,dos_, given in ppm·h, refers to the integrated concentration of NO_x_ (dose) at the total flow rate of 500 mL/min.

Figure 6a directly compares NO and NO_2_ addition and shows that both gases lead to nearly identical dielectric loss curves. This means that the same dose of NO_2_ or NO results in almost the same changes in *ε*″. The conversion of NO to NO_2_ takes place due to NO oxidation to NO_2_ in oxygen-rich atmospheres on platinum sites in the storage material, and the effects of the nitrite or nitrate species formed on the dielectric losses are almost identical, regardless of whether NO or NO_2_ is added.

A more precise dependence of the dielectric losses on the actual amount of NO_x_ stored or on the calculated proportion of occupied barium storage sites is shown in Figure 5c and Figure 5f. Here, there is also a linear relationship between *ε*″ and *w*_Ba,NOx_, whereby the influence of temperature and the added NO_x_ species (NO_2_ vs. NO) is evident. For both NO_x_ species, the amount of NO_x_ stored decreases with increasing temperature, whereby the change in the dielectric losses, here the slope of *ε*″ vs. *w*_Ba,NOx_, increases with increasing temperature from 0.00129 (300 °C) to 0.00366 (400 °C) (NO_2_) per percent storage utilization *w*_Ba,NOx_, and 0.00151 (300 °C) to 0.00395 (400 °C) (NO) per percent storage utilization *w*_Ba,NOx_. Looking at the value of calculated storage utilization *w*_Ba,NOx_, differences between NO and NO_2_ exposure become apparent. At 300 °C and 350 °C only 10.3–10.7% Ba is utilized for NO storage, whereby in the case of NO_2_ 14.9–15.5% is reached. The difference becomes smaller at 400 °C, with 8.9% (NO) and 11.5% (NO_2_).

Figure 6b highlights these differences, that presumably arise from the temperature-dependent Pt-catalyzed oxidation of NO to NO_2_. Depending on the temperature, NO is not completely oxidized to NO_2_, or the oxidation reaction and the NO:NO_2_ equilibrium are kinetically limited [64,69]. Since only NO_2_ can be stored as nitrate in the storage material, the rate of NO_2_ formation limits the storage. Importantly, the dielectric losses and their characteristic slopes remain identical regardless of whether NO or NO_2_ is supplied. Therefore, the NO_x_ storage materials are suitable for use in a RF-based dosimeter-type total NO_x_ sensor.

### 3.3. Operando DRIFTS Investigation of NSC-Ba17

**Figure 7:**
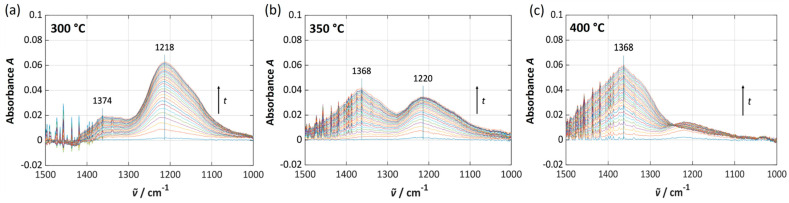
Absorbance *A* for the most relevant wave number range (from 1500 cm^−1^ to 1000 cm^−1^) obtained from DRIFTS during exposure of an NSC-Ba17 film to 2 ppm NO over 35 min at different temperatures: (**a**) 300 °C, (**b**) 350 °C, and (**c**) 400 °C.

To gain deeper insight into the storage mechanism of NSC-Ba17 and the relationship between sorbate formation and electrical response, operando DRIFTS measurements were performed. A screen-printed NSC-Ba17 film was exposed to 2 ppm NO for *t* = 35 min at 300 °C, 350 °C, and 400 °C, and the resulting sorbed species (nitrites and nitrates) were monitored. From the recorded spectra (intensity (a.u.) vs. wave number (cm^−1^)), the absorbance *A* was calculated. Figure 7 shows the resulting values in the relevant wave number region between 1500 cm^−1^ and 1000 cm^−1^. At 300 °C (Figure 7a), two adsorption bands appear at 1374 cm^−1^ and 1218 cm^−1^, both increasing in intensity with NO dosing time and resulting NO_x_ loading. According to the literature, the adsorption band at around 1374 cm^−1^ is assigned to nitrate species and the band at around 1218 cm^−1^ corresponds to nitrite species [34,70,71]. Both species, nitrites and nitrates, can be formed on alumina or on barium sites. Reported wavenumber ranges for Ba nitrites (1210–1220 cm^−1^) and Ba nitrates (e.g., 1410–1423 cm^−1^, 1314–1335 cm^−1^, 1022–1037 cm^−1^, 1480 cm^−1^ and 1130 cm^−1^) are consistent with the observed band positions [34]. According to [72], nitrates can also be found between 1375 and 1329 cm^−1^. In [70], bands in the region 1360–1300 cm^−1^ are attributed to formed ionic nitrates on Pt–Ba–Al_2_O_3_ and hypo-nitrates are responsible for bands at 1375 cm^−1^ during NO exposure. These assignments match the bands visible in Figure 7a quite well. Slight band shifts can occur due to sample preparation and the setup of the DRIFTS cell [72]. With increasing temperature to 350 °C and 400 °C (Figure 7b,c), the nitrate band at 1374–1368 cm^−1^ becomes more pronounced, while the nitrite band at 1220 cm^−1^ diminishes and nearly disappears. Two effects contribute to this behavior. First, since NO was used as test gas, it must be oxidized to NO_2_ on Pt sites before nitrate formation can occur. The kinetic of the NO oxidation accelerates with increasing temperature, enhancing nitrate formation. Second, nitrites are often formed initially as intermediates during NO_x_ storage. With increasing temperature, initially formed barium nitrites are more rapidly oxidized to nitrates [34,73]. Both effects can contribute to the observed changes in the intensity of the bands.

**Figure 8:**
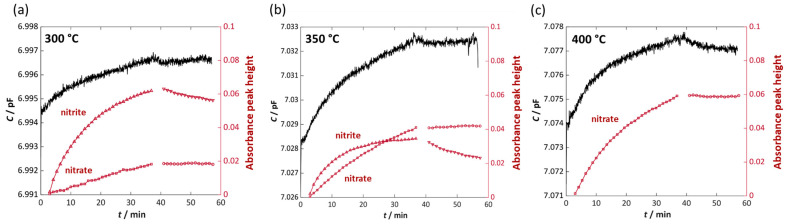
Peak height of the absorbance *A* evaluated at 1218–1220 cm^−1^ (denoted as nitrite) and 1374–1368 cm^−1^ (denoted as nitrate) and the calculated capacitance *C* during exposure of a NSC-Ba17 film to 2 ppm NO at different temperatures: (**a**) 300 °C, (**b**) 350 °C, and (**c**) 400 °C.

To better illustrate the time-dependent behavior of sorbate formation and the corresponding electrical response, Figure 8 shows the peak heights of the absorbance bands at 1218–1220 cm^−1^ (denoted as nitrite) and 1374–1368 cm^−1^ (denoted as nitrate), plotted as a function of NO dosing time. In addition, the operando-measured and calculated capacitance *C* is inserted. At 300 °C (Figure 8a), the nitrite peak height increases during NO exposure with increasing NO dose. Simultaneously, the nitrate peak height increases nearly linearly with NO dosing time. The simultaneously measured capacitance signal shows a slight increase (Δ*C* = 1.9 fF). At 40 min, the dosage of NO was stopped. A material appropriate for a NO_x_ gas dosimeter should provide good holding capabilities and the sorbed species should not be desorbed during NO_x_ dosing pauses. The results show that at 300 °C, the absorbance peak height of nitrates remains constant, demonstrating that the nitrate species are strongly bound and no desorption occurs. In contrast, a slight desorption of weakly bound nitrite species occurs, visible by a decreasing peak height of the absorbance. The capacitance shows a slight decrease when NO dosing is stopped but remains almost constant during the following NO_x_ dosing pause.

At 350 °C, the height of the nitrite peak becomes smaller whereas the nitrate peak continues to increase, confirming that higher temperatures promote nitrate formation, as discussed previously. The nitrite band reaches saturation quickly, whereas the nitrate band increases almost linearly throughout the NO exposure period. The capacitance increases more strongly than at 300 °C (Δ*C* = 4 fF), reflecting the higher fraction of nitrates formed and the associated change in dielectric permittivity *ε*′. This is consistent with the results discussed in Section 3.1 (see Figure 2 and Figure 5). After switching off the NO dosing, nitrite desorption becomes visible, while the nitrate peak remains constant. The capacitance decreases slightly at first but does not follow nitrite desorption directly, and *C* remains constant afterwards, like the nitrate peak height. At 400 °C (Figure 8c), only nitrates are detected; the nitrite band is no longer observable (Figure 7c). The nitrate peak height increases continuously with NO dose and does not reach saturation within 35 min, indicating that the material is not fully loaded. The capacitance also increases steadily (Δ*C* = 3.8 fF), reflecting ongoing nitrate formation throughout the storage phase. Here, like at 350 °C, the nitrate peak remains constant during NO_x_ dosing pause and the capacitance behaves similarly.

A comparison of radio frequency characterization of Pt-Ba-based NO_x_ storage powders with the operando DRIFTS investigations on screen-printed NSC-Ba17 films shows that both methods yield consistent trends in the material’s behavior. The RF measurements demonstrate that both permittivity *ε*′ and dielectric losses *ε*″ increase with NO_x_ loading, although a robust quantitative correlation can only be established for *ε*″. The strong temperature dependence of the resonance frequency makes the evaluation of *ε*′ difficult, whereas *ε*″ shows a clear linear relationship with the stored NO_x_ amount, expressed as the fraction of utilized Ba storage sites *w*_Ba,NOx_. The slope of the respective characteristic curves depends on temperature. Furthermore, RF data show that once the storage capacity is approached, both the stored NO_x_ amount and *ε*″ begin to level off, indicating saturation of the Ba sites. The operando DRIFTS results confirm that nitrites and nitrates are formed on the storage material during NO_x_ exposure, and their quantities increase with NO_x_ dose. At lower temperatures (300 °C), nitrogen oxides are mainly sorbed as nitrite species, whereas at higher temperatures (400 °C), only nitrate species remain stable. At the same time, a slight increase in measured capacitance was observed, attributable to changes in permittivity, and thus can be linked to the evolving dielectric properties of the NO_x_ storage material. Taken together, these findings demonstrate that the observed changes in both *ε*′ and *ε*″ originate from the formation of nitrite and nitrate species. Whether one of the formed species dominates the dielectric properties could not be clarified during the investigations.

The experiments also reveal differences in the desorption behavior. Immediately after NO_x_ dosing stops, both *ε*″ and the calculated Ba utilization decrease, and this effect becomes stronger with increasing temperature. In contrast, *ε*′ remains nearly constant during desorption and the measured capacitance, which correlates primarily with *ε*′, also remains almost constant. Operando DRIFTS measurements show that nitrates do not desorb in NO_x_ free phases, even at elevated temperatures, whereas nitrites desorb readily and less nitrite forms as the temperature increases. The measured capacitance does not display a clear signature of nitrite desorption.

These observations suggest that nitrites and nitrates influence the dielectric properties in different ways and that *ε*′ and *ε*″ may be governed by different sorbate species or mechanisms. However, the present data do not allow an unambiguous separation of their respective contributions. Additionally, the RF measurements were performed on powder samples, while the DRIFTS investigations were conducted on porous films, and the applied NO_x_ concentrations and doses differed between the experiments. This likely resulted in different NO_x_ loading states. It can be assumed that the powders reached a more complete NO_x_ loading, which may have led to increased desorption of both nitrite and nitrate species during the NO_x_-free phases.

## 4. Conclusions

This study provides a comprehensive analysis of the dielectric behavior and NO_x_ storage characteristics of Pt/Ba–Al_2_O_3_-based NO_x_ storage materials using microwave cavity perturbation (MCP), operando DRIFTS, and impedance-based measurements of functional films. Across all experiments, the dielectric losses *ε*″ were identified as the most robust and sensitive indicator of NO_x_ uptake, while the permittivity *ε*′ exhibited only minor and temperature-dependent variations. A clear linear correlation was established between *ε*″ and the amount of stored NO_x_, expressed as the fraction of occupied Ba storage sites. This linear regime is particularly relevant for RF-based dosimeter-type sensors, as it enables the cumulative quantification of NO_x_ doses.

The NO_x_ storage experiments revealed that, independent of the absolute barium loading, approximately 35% of the available Ba sites participate in the formation of nitrite and nitrate species under the applied storage conditions. However, the absolute amount of stored NO_x_ and the resulting magnitude of the dielectric loss change scale directly with the Ba content. This correlation enables a material-specific sensitivity tuning for RF-based NO_x_ dosimeters. A linear relationship between *ε*″ and both the NO_x_ dose and the calculated Ba site utilization was established for all powders within their respective linear storage regime. This linear regime is particularly relevant for dosimeter-type sensors, as it defines the region in which cumulative NO_x_ quantification is linearly possible.

Temperature-dependent experiments revealed stable NO_x_ storage and a reproducible *ε*″ response up to about 350 °C. At 400 °C, desorption becomes significant, leading to deviations from linear dosimeter behavior. Experiments with NO and NO_2_ showed nearly identical *ε*″ signatures, confirming that NO is oxidized to NO_2_ on Pt prior to storage and that the dielectric response is governed by the subsequent formation of nitrite and nitrate species.

Operando DRIFTS provided mechanistic insight into the species responsible for the dielectric response. At lower temperatures, both nitrites and nitrates are formed, whereas at higher temperatures nitrates dominate. Nitrite species desorb readily during NO_x_-free phases, while nitrates remain strongly bound. A similar dependency of the strongly and weakly bound species was described in [46]. These findings agree with the observed behavior of *ε*″, which decreases during desorption phases and increases with progressive nitrate formation. Capacitance measurements performed simultaneously with DRIFTS showed only small changes in *ε*′ and did not correlate directly with nitrite desorption, indicating that nitrates predominantly influence the dielectric properties at the operating temperatures studied.

Overall, the results demonstrate that Pt/Ba–Al_2_O_3_ NO_x_ storage materials exhibit a reproducible and chemically interpretable dielectric response to NO_x_ storage, governed by temperature-dependent formation and stability of nitrite and nitrate species. The strong and linear dependence of *ε*″ on the NO_x_ dose and Ba site utilization confirms the suitability of these materials for RF-based dosimeter-type NO_x_ sensors. The combination of MCP, DRIFTS, and impedance analyses provides a coherent mechanistic understanding of the dielectric signal formation and establishes the methodological framework for future sensor optimization.

Future work will focus on transferring the insights gained from the powder-based MCP studies and the operando investigations on functional films to the high-frequency dosimeter transducer introduced in [46]. A key goal will be to evaluate how the dielectric and sorption-related properties determined from powders and screen-printed films translate to the performance of an NSC-Ba17-coated RF dosimeter. Particular attention will be given to understanding how the temperature-dependent formation and stability of nitrites and nitrates influence sensor linearity, sensitivity, and long-term stability with respect to dosimeter-like NO_x_ detection.

## Data Availability

All relevant data presented in the article are stored according to institutional requirements and as such are not available online. However, all data used in this paper can be made available upon request to the authors.

## Abbreviations

The following abbreviations are used in this manuscript:

NSC	NO_x_ storage catalyst
RF	Radio frequency
MCP	Microwave Perturbation Method
DRIFTS	Diffuse Reflectance Infrared Fourier Transformed Spectroscopy
FTIR	Fourier Transformed Infrared Spectroscopy
ppm	Parts per million
CLD	Chemiluminescence detector
